# Suboptimal management of hypercholesterolemia in countries with high or very high cardiovascular risk: findings from the international DISCOVERY study

**DOI:** 10.3389/fcvm.2025.1665059

**Published:** 2025-09-05

**Authors:** Mišo Šabović, Hristo Pejkov, Alexandru Caraus, Ivan Gruev, Vlad Damian Vintilă, Zoltán Csanádi, Sodgerel Batjargal, Tamara Kovačević-Preradović, Zumreta Kušljugić, Draško Kuprešak, Zaim Jatić, Gani Bajraktari

**Affiliations:** ^1^ Clinical Department of Vascular Diseases, University Medical Centre Ljubljana, Ljubljana, Slovenia; ^2^ Medical Faculty, University of Ljubljana, Ljubljana, Slovenia; ^3^ Medical Faculty Skopje, University Clinic of Cardiology, St. Cyril and Methodius University, Skopje, North Macedonia; ^4^ National Cardiology Institute, Chisinau, Moldova; ^5^ National Transport Hospital Tzar Boris III, Sofia, Bulgaria; ^6^ Cardiology Department, Carol Davila University of Medicine and Pharmacy, University and Emergency Hospital, Bucharest, Romania; ^7^ Cardiology and Heart Surgery Clinic, University of Debrecen, Debrecen, Hungary; ^8^ Department of Cardiovascular Surgery, Institute of Medical Sciences, Mongolian National University of Medical Sciences, Ulaanbaatar, Mongolia; ^9^ Department of Cardiology, University Clinical Center of the Republic of Srpska, Banja Luka, Bosnia and Herzegovina; ^10^ Kardio Centar Prof. dr. Zumreta Kušljugić, Tuzla, Bosnia and Herzegovina; ^11^ Family Medicine, Health Centre Čelinac, Čelinac, Bosnia and Herzegovina; ^12^ Department of Family Medicine, Faculty of Medicine, University of Sarajevo, Sarajevo, Bosnia and Herzegovina; ^13^ Medical Faculty, University of Prishtina Hasan Prishtina, University Clinical Centre of Kosova, Prishtina, Kosovo

**Keywords:** LDL-C, hypercholesterolemia, lipid-lowering therapy, ESC/EAS guidelines, cardiovascular risk, real-world evidence, statins, ezetimibe

## Abstract

**Introduction:**

The 2019 ESC/EAS guidelines introduced stricter low-density lipoprotein cholesterol (LDL-C) targets, particularly for patients at high and very high cardiovascular (CV) risk. However, data on the implementation of these targets in real-world clinical practice—especially in countries with high/very high CV risk—remain limited. The DISCOVERY study aimed to assess LDL-C management, lipid-lowering therapy (LLT) use, and guideline adherence across multiple countries in Central and Eastern Europe and Central Asia.

**Methods:**

This prospective, observational, multicenter study enrolled adult patients with hypercholesterolemia (HCL) from 10 countries grouped into three regions. Data was collected at baseline and after 12 weeks of follow-up. LLT patterns, LDL-C levels, target attainment (both investigator-defined and 2019 ESC/EAS-recommended), and physician adherence to guidelines were analyzed.

**Results:**

A total of 6,447 patients were included; 53.2% were female, and the mean age was 60.5 ± 11.9 years. Most patients (66%) were in secondary prevention. At baseline, 36.8% had been treated with LLT. After the first visit, treatment was changed in 78% of patients, but only 42.4% received high-intensity statins and 9.3% received statin-ezetimibe combinations at follow-up. LDL-C target achievement was poor: only 5.6% of patients met the guideline-recommended LDL-C goals, compared to 45.5% who met physician-defined targets. Among patients with ASCVD, only 3.3% achieved guideline LDL-C targets. The most significant gap was observed between guideline recommendations and physician-set LDL-C goals. No significant difference in LDL-C target attainment was observed between specialists and general practitioners.

**Discussion:**

The DISCOVERY study reveals suboptimal LDL-C control and low adherence to the 2019 ESC/EAS guidelines in routine practice across countries with high/very high CV risk. These findings highlight the urgent need for strategies to improve physician awareness, promote intensive LLT use, and close the gap between guidelines and clinical practice. A paradigm shift toward proactive LDL-C management is essential to reduce residual CV risk in these populations.

## 1 Introduction

Cardiovascular disease (CVD) has been the leading cause of death and a major cause of disability worldwide for decades (1). Since 1990, age-standardized mortality rates from CVD have declined globally, but this decline has slowed in recent years and varies considerably across regions (1, 2). High-income countries have seen more rapid improvements compared to low- and middle-income countries, where over 80% of CVD deaths occur (3). Atherosclerosis remains the principal underlying cause of CVD, driving events such as myocardial infarction and stroke (4). Effective prevention of atherosclerotic cardiovascular disease (ASCVD) requires a comprehensive, risk-based approach spanning primary and secondary care.

Among modifiable risk factors, low-density lipoprotein cholesterol (LDL-C) plays a central causal role in the development of atherosclerosis (5, 6). Therefore, LDL-C reduction is the cornerstone of ASCVD prevention (6). The 2019 guidelines from the European Society of Cardiology and the European Atherosclerosis Society (ESC/EAS) recommend intensive lowering of LDL-C, particularly in patients at very high cardiovascular risk (7, 8). These guidelines emphasize achieving a reduction of at least 50% from baseline LDL-C levels and reaching individualized LDL-C targets.

Despite these recommendations, registry and cohort studies reveal that many high-risk patients do not reach even the previous, less stringent LDL-C targets (<1.8 mmol/L or <70 mg/dl) (9–11). This gap between guidelines and real-world practice persists across Europe and has widened following the 2019 update (10–13). Importantly, cardiovascular risk and treatment success vary not only by individual patient factors but also by country of residence. The recent Santorini study, covering 14 Central and Western European countries categorized as low or moderate risk, reported only 20.1% of patients achieving LDL-C targets (11). Comparable data for high and very high-risk countries are lacking.

The international DISCOVERY study was designed to fill this knowledge gap by prospectively evaluating the management of hypercholesterolemia and arterial hypertension (hypertension data not shown here) in high and very high cardiovascular risk countries. The primary goal was to provide up-to-date real-world data on the achievement of 2019 ESC/EAS LDL-C targets across patient groups (ASCVD, diabetes, and apparently healthy individuals). These findings aim to inform clinical practice and improve cardiovascular outcomes in high-risk populations.

## 2 Methods

### 2.1 Study design

The DISCOVERY study was an international, multicenter, prospective, observational cohort study designed to evaluate the management of hypercholesterolemia (HCL) and arterial hypertension in routine clinical practice. This article focuses exclusively on the analysis and discussion of data from patients with HCL. Between March 2021 and December 2022, a total of 6,447 patients with HCL were enrolled from primary and secondary care centers across multiple countries. Patients were distributed across three regions: 14.5% (*n* = 932) from Region 1, 77.2% (*n* = 4,976) from Region 2, and 8.4% (*n* = 539) from Region 3, with further details provided in Table 1.

**Table 1:** The number of patients included diagnosed with hypercholesterolemia and routinely treated by country and region.

Region/country	Included patients
Region 1, *n* (%)	932 (14.1)
Hungary	454 (7.0)
Slovenia	478 (7.4)
Region 2, *n* (%)	4,976 (77.2)
Bosnia and Herzegovina	1,036 (16.1)
Bulgaria	1,308 (20.3)
Kosovo	229 (3.6)
North Macedonia	1,166 (18.1)
Romania	1,237 (19.2)
Region 3, *n* (%)	539 (8.4)
Moldova	89 (1.4)
Mongolia	76 (1.2)
Uzbekistan	374 (5.8)
Total	6,447 (100.0)

The study involved 772 investigators, including general practitioners (39.0%), cardiologists (35.1%), internists (16.5%), and other specialists (9.5%). Prior to enrollment, all patients received comprehensive verbal explanations of the study procedures from the investigators, presented in clear and understandable language. Additionally, patients were provided with written information in their native language.

Data collection was conducted exclusively on patients who provided informed consent for the use, analysis, and disclosure of their personal data in compliance with the European General Data Protection Regulation (GDPR, Regulation 2016/679). The study was carried out in accordance with the ethical standards of the Declaration of Helsinki, good pharmacovigilance practice, and the applicable national legislation governing epidemiological research in each participating country. The study did not interfere with the standard care practice, and any follow-up procedures were not part of this study. The study protocol was reviewed and approved by the Ethics Committees of all participating countries, with documented approvals obtained in accordance with local regulations

### 2.2 Eligibility criteria

Adults aged 18 years and older with hypercholesterolemia (HCL), either newly diagnosed or previously treated but uncontrolled on existing lipid-lowering therapy (LLT), were eligible for inclusion. Newly diagnosed patients were defined as those who had never received LLT or had discontinued LLT for at least two months prior to study enrollment. Key exclusion criteria included hypersensitivity to any active ingredients or components of the LLT, contraindications listed in the LLT prescribing information, and female participants who were pregnant, breastfeeding, or planning pregnancy.

### 2.3 Data collection

Prior to study initiation, investigators (detailed in Supplementary Table S1) completed a questionnaire assessing their approach to HCL management. Responses from 772 investigators were collected, providing insight into clinical practice patterns. Patients were followed prospectively over a 12-week period with data collected at two visits. The first visit coincided with patient enrollment, while the second visit occurred up to 12 weeks thereafter. Data were captured via a standardized electronic case report form (eCRF), and all visits adhered to routine clinical practice guidelines within each participating country.

At the first visit, demographic information, smoking status, physical activity, cardiovascular risk factors, history of CVD events, HCL diagnosis details, concurrent treatments and investigator-defined LDL-C target were recorded. The second visit focused on documenting HCL treatment regimens, any modifications to concomitant therapies, and adverse events occurring between visits.

### 2.4 Aims and outcomes

The primary objective of the DISCOVERY study's hypercholesterolemia (HCL) component was to collect and compare real-world data on HCL management across participating countries from different regions, thereby enhancing understanding of local clinical practices. The main outcome measure was the proportion of patients within predefined subgroups (7, 8) (see Supplementary Figure S1) achieving target low-density lipoprotein cholesterol (LDL-C) levels at the second visit, in line with the 2019 ESC/EAS guidelines (7) (Supplementary Table S2).

Participants categorized as apparently healthy—defined as individuals without established atherosclerotic cardiovascular disease (ASCVD), diabetes mellitus (DM), chronic kidney disease (CKD), or familial hypercholesterolemia (8)—were classified as primary prevention patients. Their 10-year cardiovascular risk was estimated using the Systematic Coronary Risk Evaluation (SCORE) and stratified as low, moderate, high, or very high according to the 2019 ESC/EAS guidelines (7). Among patients with specific risk conditions (8), only those with DM were included and are hereafter referred to as the diabetes subgroup. Their cardiovascular risk was assessed and categorized as moderate, high, or very high per the 2019 ESC/EAS guidelines and the 2021 ESC guidelines on CVD prevention in clinical practice (7, 8). All patients with established ASCVD were classified as secondary prevention patients and considered very high risk (7, 8).

As a secondary outcome, the study assessed the proportion of patients in the various subgroups (Supplementary Figure S1) who attained LDL-C target levels (Supplementary Table S2) at the second visit, with comparisons made across participating countries and regions. Additionally, patterns of lipid-lowering therapy (LLT) use—including type and dosage—and reasons for medication changes were evaluated. Given that the study preceded the publication of the 2021 ESC guidelines on CVD prevention, analyses were conducted according to LDL-C targets recommended in the 2019 ESC/EAS guidelines (Supplementary Figure S1).

### 2.5 Statistical analysis

All statistical analyses were conducted by a qualified biomedical statistician. Descriptive statistics were used to summarize the data. For categorical variables, results were presented as absolute frequencies and corresponding percentages. Due to rounding, the sum of percentages across categories may not always total exactly 100%. For continuous variables, standard descriptive measures were reported, including the number of observations (*n*), mean, median, standard deviation (SD), minimum, maximum, and interquartile range (Q1–Q3).

Comparisons between two subgroups for variables on a ratio scale were performed using the homoscedastic (equal variance) *t*-test. For comparisons across three or more subgroups, analysis of variance (ANOVA) was employed, followed by Fisher's *post hoc* pairwise *t*-tests to assess between-group differences. For ordinal variables, the Wilcoxon–Mann–Whitney test was used for two-group comparisons, and the Kruskal–Wallis test was applied for comparisons across three groups. For nominal variables, group differences were assessed using Pearson's chi-square test. Pairwise comparisons of mean values for variables on a ratio scale were conducted using standard *Z*-tests. A multiple linear regression model was fitted to the data, with overall model significance evaluated using the *F*-test and the significance of individual predictors assessed via *t*-tests for regression coefficients. All analyses were performed in R using the *lm* function. Statistical significance was defined as a two-tailed *p*-value < 0.05.

## 3 Results

### 3.1 Baseline demographic and clinical characteristics

**Figure 1:**
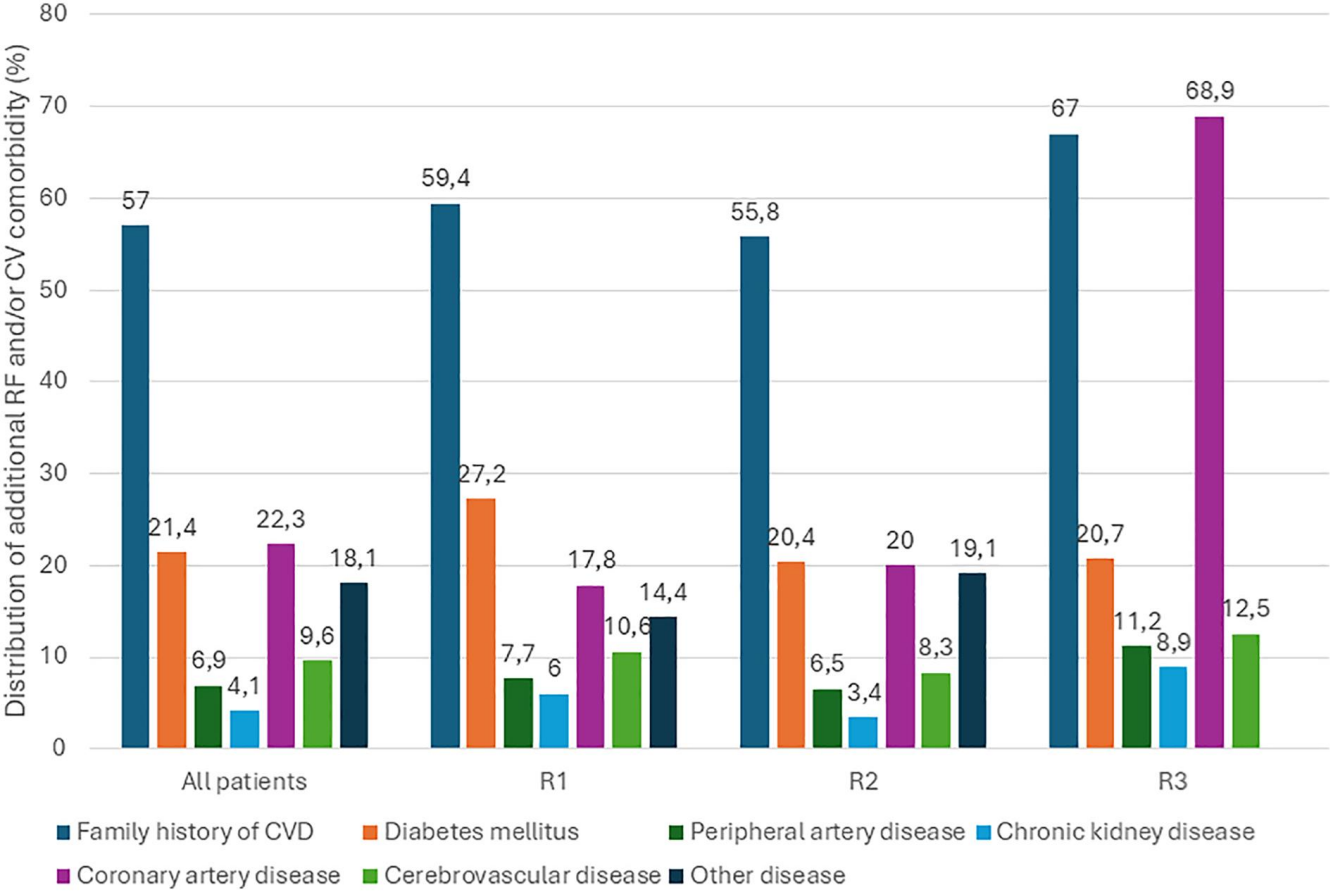
Distribution of concomitant diseases overall and by region. R, region; RF, risk factor; CV, cardiovascular*.*

Baseline demographic and clinical characteristics of the entire DISCOVERY study population are presented in Table 2. The mean age of patients was 60.5 ± 11.9 years, with women comprising 53.2% of the total cohort. Overall, 77% of participants presented with at least one additional cardiovascular (CV) risk factor or comorbidity. The most commonly reported comorbid condition was a family history of cardiovascular disease (57.0%), followed by coronary artery disease (22.3%) and diabetes mellitus (21.4%). As patients could report multiple conditions, overlaps between comorbidities were common. A detailed breakdown of comorbidities—both overall and by region—is shown in Figure 1. In terms of multimorbidity, the largest subgroup included patients with one concomitant disease (*n* = 4,303; 38.1%), followed by those with two concomitant diseases (*n* = 2,701; 23.9%) and those with four or more concomitant conditions (*n* = 609; 5.4%). Significant sex-related differences were observed. Women were older (63.4 vs. 59.9 years) and had higher baseline LDL-C (3.93 vs. 3.80 mmol/L; both *p* < 0.001). Men had higher BMI (29.1 vs. 28.9 kg/m², *p* = 0.029), blood pressure (154.5/92.1 vs. 153.5/90.8 mmHg; *p* ≤ 0.037), higher rates of current (31% vs. 18%) and former smoking (26% vs. 10%; both *p* < 0.001), and more frequent physical activity (1.0 vs. 0.83, *p* < 0.001).

**Table 2:** Baseline demographics and patient characteristics.

Characteristics	Overall (*n* = 11,287)
Female, *n* (%)	6,009 (53.2)
Age, years, average (SD)	60.5 (11.9)
AH only, *n* (%)	4,191 (37.1)
HCL only, *n* (%)	723 (6.4)
AH and HCL, *n* (%)	6,373 (56.5)
Height, cm, average (SD)	169.5 (8.8)
Weight, kg, average (SD)	82.7 (15.2)
BMI, kg/m^2^, average (SD)	28.7 (4.6)
Smoking history, *n* (%)	
Current	2,817 (25)
Former	1,857 (16)
Never	6,613 (59)
Physical activity, *n* (%)	
1–2 times a week	4,733 (42)
3–5 times a week	1,806 (16)
>5 times a week	613 (5)
Not physically active	4,135 (37)
Additional risk factors or concomitant diseases^a^, *n* (%)	
Family history of CVD	6,430 (57.0)
Coronary artery disease	2,518 (22.3)
Diabetes	2,421 (21.4)
Cerebrovascular disease	1,084 (9.6)
Peripheral artery disease	779 (6.9)
Chronic kidney disease	463 (4.1)
Other	2,039 (18.1)

AH, arterial hypertension; HCL, hypercholesterolemia; BMI, body mass index; CVD, cardiovascular disease; SD, standard deviation.

^a^ Each patient could have several diseases; the table reflects data from patients across the entire DISCOVERY study.

### 3.2 Achievement of LDL-C targets

In the DISCOVERY study, LDL-C target attainment was assessed both according to individualized targets set by investigators at baseline (first visit) and in accordance with the 2019 ESC/EAS guideline recommendations. Changes in lipid parameters and the proportion of patients achieving these targets were analyzed by patient subgroup.

Out of 6,447 patients enrolled, 4,136 had LDL-C values recorded at both the first and second visits along with complete clinical data. Among them, 6% were classified as primary prevention patients, 28% had diabetes mellitus, and 66% were categorized as secondary prevention patients (14). Across the total population, 45.5% (*n* = 2,045) achieved the LDL-C targets defined by their treating physician. However, only 5.6% (*n* = 230) of patients met the guideline-recommended LDL-C goals, including both absolute and relative reductions as specified in the 2019 ESC/EAS guidelines (14).

**Figure 2:**
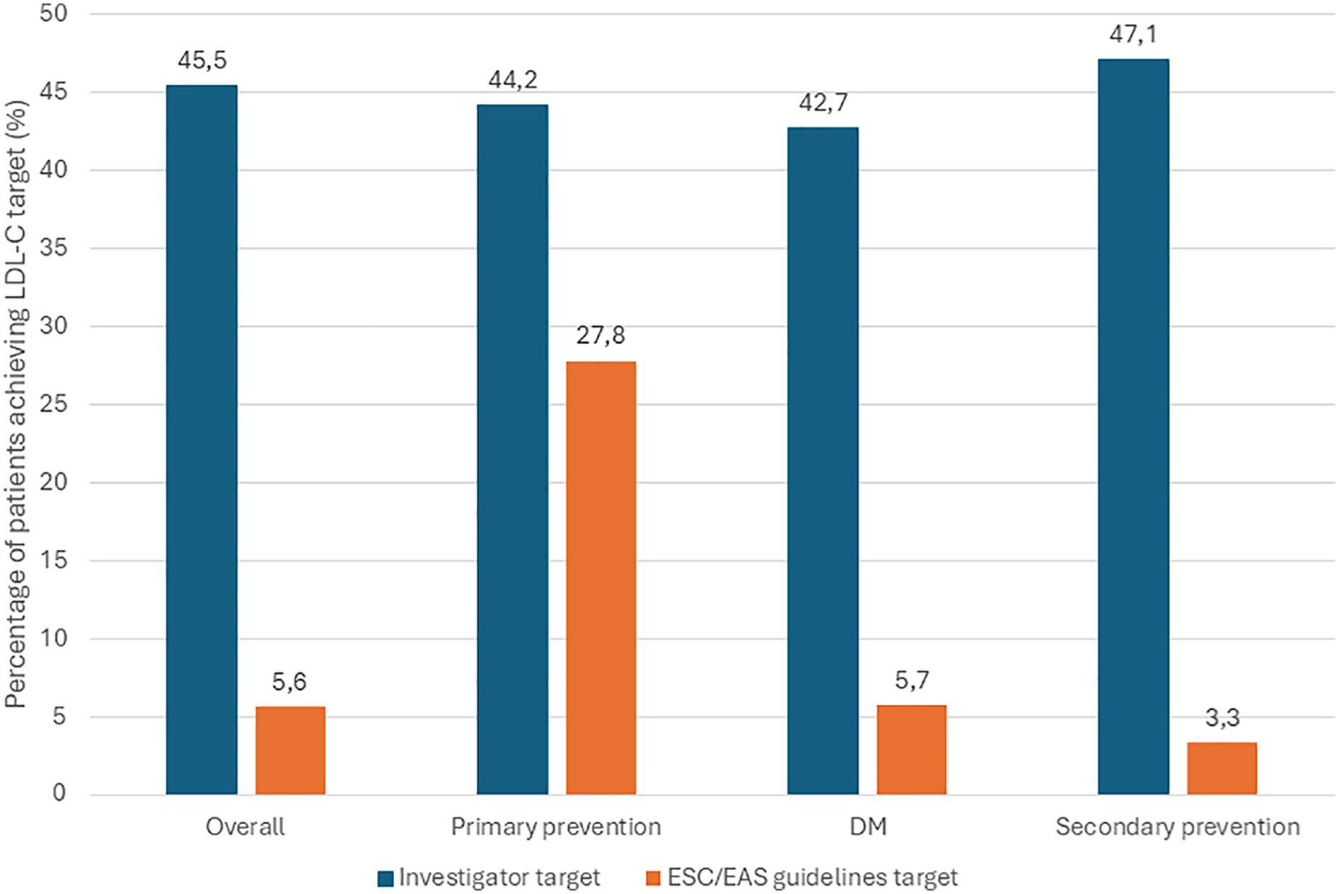
Comparison between the achievement of target LDL-C levels by investigator and by 2019 ESC/EAS guidelines (overall and by category of individuals) at 2nd visit. HCL, hypercholesterolemia; LDL-C, LDL cholesterol; LLT, lipid-lowering therapy; ESC/EAS, European Society of Cardiology/European Atherosclerosis Society; DM, diabetes mellitus. * Percentages with respect to those patients with HCL diagnosis and on LLT who had both LDL-C level record at 2nd visit and the record of the investigator-determined LDL-C target level. ** Percentages with respect to those HCL patients on LLT who had records of LDL-C level at 2nd visit and for whom it was possible to determine their target LDL-C according to the ESC/EAS guidelines.

A multiple linear regression analysis was performed on patients (*n* = 4,136) with available LDL-C change data and complete clinical data, incorporating age, sex, baseline LDL-C, and ASCVD status as predictors of absolute LDL-C change. Greater absolute LDL-C reductions were significantly associated with higher baseline LDL-C (*β* = −0.69, *p* < 2 × 10⁻¹⁶), male sex (*β* = −0.071, *p* = 0.0023), and ASCVD (*β* = −0.082, *p* = 1.11 × 10⁻⁶). Age was not a significant predictor (*β* = −0.00052, *p* = 0.635).Target attainment varied by subgroup: 27.8% (*n* = 74) of primary prevention patients reached the guideline-defined LDL-C goal, compared to 5.7% (*n* = 66) of patients with diabetes and just 3.3% (*n* = 90) of secondary prevention patients. A detailed comparison between LDL-C goal achievement based on investigator-defined and guideline-based targets is shown in Figure 2. These findings highlight a marked discrepancy between routine clinical targets and evidence-based recommendations.

**Figure 3:**
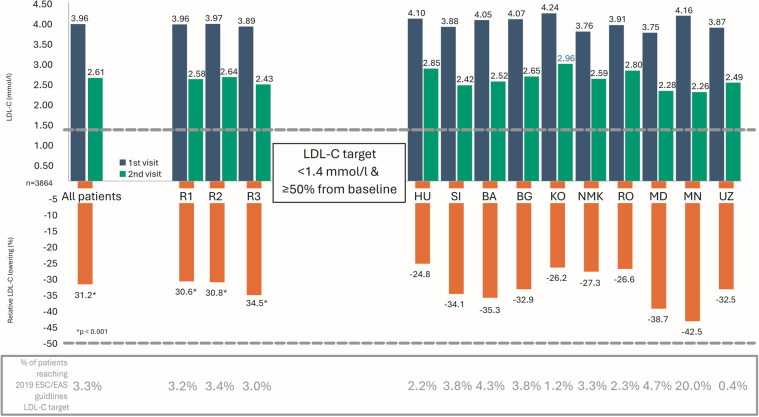
Comparison between regions and countries in terms of LDL-C lowering (1st and 2nd visits) and in terms of the achievement of LDL-C target recommended by 2019 ESC/EAS guidelines at 2nd visit among secondary prevention patients. LDL-C, LDL cholesterol; ESC/EAS, European Society of Cardiology/European Atherosclerosis Society; R, region; HU, Hungary; SI, Slovenia; BA, Bosnia and Herzegovina; BG, Bulgaria; KO, Kosovo; NMK, North Macedonia; RO, Romania; MD, Moldova; MN, Mongolia; UZ, Uzbekistan.

The investigator-defined LDL-C target for all patients was 2.33 ± 0.65 mmol/L. In patient subgroups, the targets were as follows: primary prevention, 2.52 ± 0.64 mmol/L; diabetes, 2.17 ± 0.64 mmol/L; and ASCVD, 2.35 ± 0.64 mmol/L. The proportion of patients achieving investigator-defined LDL-C targets was similar across subgroups. Regional differences in target attainment were minor and mostly non-significant, except for statistically significant differences between Region 2 and Region 3 among all patients, and within the primary and secondary prevention groups (*p* < 0.001). Differences between regions in achieving LDL-C targets per 2019 ESC/EAS guidelines were also small and clinically non-relevant, including among secondary prevention patients with the highest cardiovascular risk. Detailed interregional and country-level comparisons of LDL-C reduction (from Visit 1 to Visit 2) and target achievement in secondary prevention patients are shown in Figure 3.

The DISCOVERY study was conducted across various clinical settings, involving 772 investigators: 39% general practitioners (GPs), 35.1% cardiologists, 16.5% internists, and 9.5% other specialists. GPs were most represented in Region 1 and in Romania (Region 2), while in other countries patients were predominantly treated by specialists. To assess whether physician specialty influenced HCL management, a subgroup analysis was performed. Patients were stratified by ASCVD status (secondary prevention vs. non-ASCVD) and by physician specialty (GP vs. specialist).

Among patients with ASCVD, LDL-C levels significantly declined from the first to the second visit (*p* < 0.001), with significant reductions observed across all subgroups (overall, specialists, and GPs). However, no statistically significant difference in the mean LDL-C reduction was found between patients treated by specialists and those treated by GPs.

In contrast, among non-ASCVD patients, a significantly greater LDL-C reduction was observed in those treated by specialists compared to those managed by GPs. LDL-C levels and changes by specialty and prevention group are summarized in Table 3.

**Table 3:** LDL-C values and differences (overall and by specialization).

Group	Patients with diagnosed HCL at 2nd visit	LDL-C (mmol/L)					
		Patients with values at both captures	Value at visit 1	Value at visit 2	Mean absolute difference	Mean relative difference	*p*-value of paired comparison
ASCVD patients	4,103	2,732 (66.6%)	3.96 (±1.05)	2.61 (±0.82)	−1.35 (±1.04)	−31.1% (±23.6%)	<0.001
* *ASCVD specialists	2,585	1,776 (68.7%)	3.93 (±1.06)	2.54 (±0.79)	−1.39 (±1.02)	−32.4% (±23.3%)	<0.001
* *ASCVD GPs	1,518	956 (63%)	4.01 (±1.02)	2.74 (±0.85)	−1.27 (±1.07)	−28.8% (±23.9%)	<0.001
Comparison of mean absolute differences among ASCVD specialists and GPs; *p*-value							0.003
Non-ASCVD patients	2,758	1,859 (67.4%)	3.95 (±1.16)	2.63 (±0.89)	−1.32 (±1.13)	−29.6% (±26.1%)	<0.001
Non-ASCVD pecialists	1,655	1,173 (70.9%)	3.98 (±1.18)	2.57 (±0.86)	−1.41 (±1.17)	−31.6% (±25.6%)	<0.001
* *Non-ASCVD GPs	1,103	686 (62.2%)	3.89 (±1.12)	2.74 (±0.92)	−1.16 (±1.03)	−26.3% (±26.8%)	<0.001
Comparison of mean absolute differences among non-ASCVD specialists and GPs; *p*-value							<0.001

LDL-C, LDL cholesterol; HCL, hypercholesterolemia; ASCVD, atherosclerotic cardiovascular disease; GP, general practitioner.

LDL-C target achievement was assessed in 4,129 patients with complete data, including 2,720 managed by specialists and 1,409 by GPs. Of these, 66% (*n* = 2,708) were ASCVD patients. Among secondary prevention patients, only 3.3% (*n* = 90) met the 2019 ESC/EAS LDL-C targets: 3.8% (*n* = 67) treated by specialists and 2.5% (*n* = 23) treated by GPs, with no significant difference between groups (*p* = 0.080). Among non-ASCVD patients, 9.9% (*n* = 140) achieved guideline-recommended LDL-C targets: 10.7% (*n* = 100) under specialist care and 8.3% (*n* = 40) under GP care, again with no statistically significant difference (*p* = 0.149).

### 3.3 Use of lipid-lowering therapy (LLT)

Among the 6,447 patients with hypercholesterolemia (HCL) enrolled in the study, 36.8% (*n* = 2,372) were already receiving lipid-lowering therapy (LLT) prior to the first study visit. At baseline, treatment modifications were made in 78% of all patients. Specifically, a new LLT was introduced in 88.6% (*n* = 5,712), a new single-pill combination (SPC) was prescribed in 9.2% (*n* = 593), 16.8% (*n* = 1,083) had prior therapy discontinued or changed due to inefficacy or safety concerns, and 8.7% (*n* = 561) had their current dose adjusted upward.

At the second visit, among 6,245 treated patients, 42.4% (*n* = 2,648) were receiving high-intensity statins, 47.1% (*n* = 2,941) were treated with low- to moderate-intensity statins, and 9.3% (*n* = 581) were on combination therapy with a statin and ezetimibe. High-intensity statins in this context refer to regimens expected to reduce LDL-C levels by ≥50%, while moderate-intensity therapies correspond to a reduction of approximately 30% (15).

**Figure 4:**
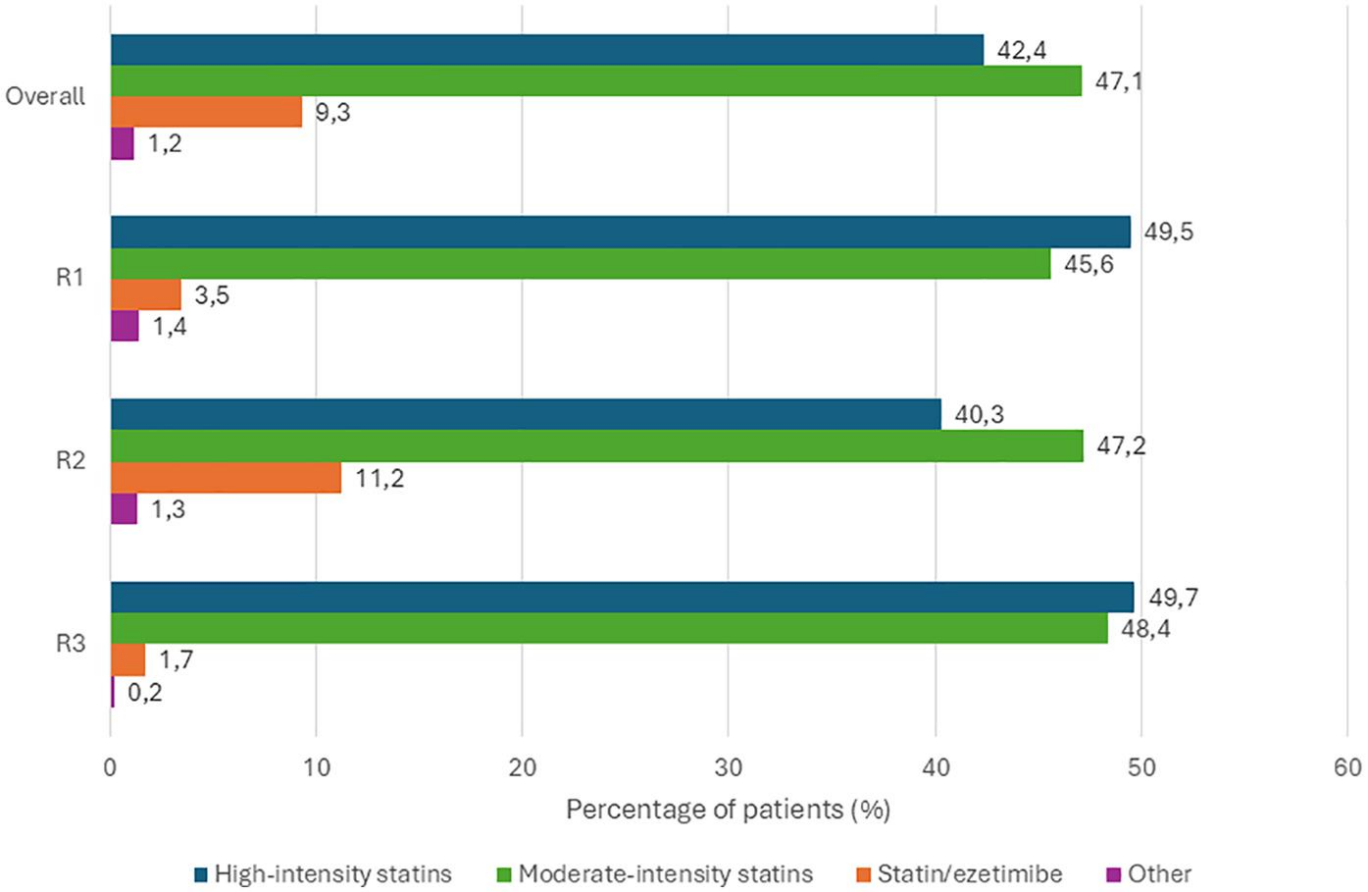
Modified version of the continuing LLT from 1st to 2nd visit overall and by region, adapted from Šabović et al. (14). R, region; PCSK9, proprotein convertase subtilisin/kexin type 9; LLT, lipid-lowering therapy. * fenofibrate, ezetimibe, omega-3 products, red yeast rice products, simvastatin/fenofibrate, PCSK9 inhibitors, artichokes product, ciprofibrate.

Patients with LDL-C values recorded at both visits showed a statistically significant reduction in LDL-C levels (*p* < 0.001). More detailed data on LLT continuation and LDL-C reduction are provided in Table 4; Figure 4.

**Table 4:** LDL-C values and differences from 1st to 2nd visit (by used LLT).

	No. of treated patients	LDL-C (mmol/L)					
		Patients with values at both captures	Value at visit 1	Value at visit 2	Mean absolute difference	Mean relative difference	*p*-value of paired comparison
On high-intensity statins	2,734	2,002 (73.2%)	4.04 (±1.16)	2.58 (±0.86)	−1.46 (±1.11)	−32.5% (±25.8%)	<0.001
On moderate-intensity statins	3,036	1,935 (63.7%)	3.84 (±0.98)	2.65 (±0.80)	−1.20 (±0.96)	−28.4% (±22.4%)	<0.001
On statin and ezetimibe combination	598	447 (74.7%)	4.14 (±1.18)	2.46 (±0.92)	−1.67 (±1.13)	−38.1% (±21%)	<0.001

79 patients were treated with other LLT: fenofibrate, ezetimibe, omega-3 products, red yeast rice products, simvastatin/fenofibrate, PCSK9 inhibitors, artichokes products, ciprofibrate.

LDL-C, LDL cholesterol; LLT, lipid-lowering therapy; HCL, hypercholesterolemia.

Regional treatment patterns revealed some differences. In Region 2, 47.2% (*n* = 2,350) of patients received moderate-intensity statins, 40.3% (*n* = 2,005) high-intensity statins, and 11.2% (*n* = 556) statin-ezetimibe combination therapy. In Region 1, 49.5% (*n* = 461) were prescribed high-intensity statins, 45.6% (*n* = 425) moderate-intensity, and 3.5% (*n* = 33) combination therapy. In Region 3, 49.7% (*n* = 268) of patients were on high-intensity statins, 48.4% (*n* = 261) on moderate-intensity, and 1.7% (*n* = 9) on statin-ezetimibe therapy (14).

Among patients continuing LLT at the second visit, 5.9% (*n* = 382) were prescribed a new medication, 2.1% (*n* = 136) were started on a new SPC, and 6.6% (*n* = 432) had their current dose increased. The five most frequently prescribed LLTs at different study phases (prior to Visit 1, between Visits 1 and 2, and following Visit 2) are listed in Table 5.

**Table 5:** Top 5 prescribed LLTs before 1st visit, from 1st to 2nd visit and after 2nd visit in the AH + HCL patient subgroup.

Treatment	No. of AH + HCL patients on HCL therapy	% of treated patients^a^	Average total daily dose (mg)
Previous therapy	2,502		
Rosuvastatin	1,185 (1,182)	47.4%	15.81
Atorvastatin	1,027 (1,023)	41.0%	20.22
Simvastatin	150	6.0%	19.17
Fenofibrate	99	4.0%	163.88
Rosuvastatin/ezetimibe	64	2.6%	13.05/10
Continuing therapy between 1st and 2nd visit	5,964		
Rosuvastatin	3,880 (3,876)	65.1%	17.31
Atorvastatin	1,486 (1,481)	24.9%	24.84
Rosuvastatin/ezetimibe	469 (464)	7.9%	14.84/10
Fenofibrate	167	2.8%	159.96
Ezetimibe	96 (94)	1.6%	10
Continuing therapy from 2nd visit onwards	5,829		
Rosuvastatin	3,875 (3,862)	66.5%	18.62
Atorvastatin	1,450 (1,446)	24.9%	28.1
Rosuvastatin/ezetimibe	570 (562)	9.8%	16.87/10
Ezetimibe	175 (172)	3.0%	10
Fenofibrate	165 (164)	2.8%	174.27

LLT, lipid-lowering therapy; AH, arterial hypertension; HCL, hypercholesterolemia.

^a^ Each patient can have multiple therapies; numbers in parentheses, when present, indicate the number of patients taken into account for the computation of the average total daily dose, in case some doses’ records were disregarded.

Adherence to LLT was assessed based on investigator-reported responses from patients. Overall, 72.3% (*n* = 4,662) reported taking their LLT daily. An additional 14.1% (*n* = 906) reported missing a dose less than once per week, 4.8% (*n* = 312) reported missing 1–2 doses per week, and 2.6% (*n* = 169) missed medication more than twice weekly. For 398 patients (6.2%), adherence data were not available. It is important to note that self-reported adherence is typically overestimated in clinical research.

## 4 Discussion

The results of the DISCOVERY study, which investigated the management of hypercholesterolemia (HCL) in real-world clinical practice across 10 European and Central Asian countries between 2021 and 2022, highlight a substantial gap between the 2019 ESC/EAS guideline recommendations and their implementation in routine clinical care. This prospective observational study demonstrated that LDL-C levels remained suboptimal after 12 weeks of follow-up and, in fact, were worse than those reported in earlier studies (9–11, 16–18). Several contributing factors were identified: (i) underuse of high-intensity statins and combination therapy with ezetimibe—even in patients with established ASCVD; (ii) marked discrepancies between LDL-C targets set by treating physicians and guideline-recommended targets; and (iii) likely poor medication adherence, though not definitively measured.

Unlike most prior studies, DISCOVERY included a diverse set of countries with high or very high cardiovascular (CV) risk, grouped into three regions. Investigators collected data at baseline and after 12 weeks, reflecting their routine clinical practice. Despite reporting that guidelines were the main factor influencing treatment decisions, physicians consistently set less stringent LDL-C targets than those recommended. Consequently, while 45.5% of patients reached investigator-defined LDL-C targets, only 5.6% achieved those defined by the 2019 ESC/EAS guidelines (14). Changes in treatment were made in 78% of patients after the first visit, but they proved insufficient. At follow-up, only 42.4% of patients received high-intensity statins, 47.1% moderate-intensity statins, and 9.3% were on statin-ezetimibe combinations (14).

The regression results indicate that patients with higher baseline LDL-C, male sex and ASCVD achieve more pronounced LDL-C reductions with LLT. The lack of age influence suggests consistent treatment efficacy across age groups. These findings underscore the relevance of baseline LDL-C levels, sex, and ASCVD in modulating the lipid-lowering response. In addition, our study highlights significant sex-based differences, with women showing older age and higher LDL-C, and men higher BMI, blood pressure, smoking, and physical activity, underscoring the need for sex-specific dyslipidemia management.

Target achievement varied by risk group: LDL-C goals were reached in 27.8% of primary prevention patients, 5.7% of patients with diabetes, and only 3.3% of patients with ASCVD. Despite statistically significant LDL-C reductions across groups, these were not enough to meet the guideline-recommended targets.

These findings align with and, in some cases, worsen trends observed in other studies. The Euroaspire V study (2016–2017) found that only 29% of patients achieved an LDL-C <1.8 mmol/L despite receiving high-intensity LLT more often than moderate- or low-intensity therapy (9, 19). The Da Vinci study (2017–2018) reported LDL-C target achievement in 33% of patients under the updated 2019 ESC/EAS guidelines, with 17% and 22% target attainment in very high-risk patients in primary and secondary prevention, respectively (7, 10). The Santorini study (2020–2021) revealed that 80% of patients failed to achieve their recommended LDL-C target (3, 7). The Teresa study showed that diabetes was a positive predictor of LDL-C goal attainment; nevertheless, nearly 54% of patients with diabetes failed to achieve their LDL-C targets (7, 20).

Importantly, the DISCOVERY study tracked outcomes at two time points, unlike the cross-sectional Da Vinci and Euroaspire studies or the Santorini study, which had a 12-month follow-up (11). DISCOVERY included patients across all risk categories, improving representativeness and providing a broader view of real-world LLT practices. Moreover, it involved several countries (HU, KO, MD, MK, MN, UZ) that have not participated in similar epidemiological studies before. Given that CVD risk varies significantly between countries, especially between high- and low-income regions, this adds value and novelty to the DISCOVERY study's dataset.

Compared to previous cohorts, DISCOVERY included a higher proportion of women [53.2%; vs. 25.7% in Euroaspire V (9), 42% in Da Vinci (10), and 27.4% in Santorini (11)] and a slightly younger population (mean age 60.5 ± 11.9 years; median 62). This is notable since life expectancy in many participating countries is lower than in Western Europe.

The 2019 ESC/EAS guidelines recommend both absolute LDL-C targets (<2.6 mmol/L for moderate risk, <1.8 for high risk, and <1.4 for very high risk) and a ≥50% reduction from baseline (7, 10). While Da Vinci performed a *post-hoc* analysis of these targets, it could not evaluate relative LDL-C reduction (10). In contrast, DISCOVERY explicitly assessed both absolute and relative LDL-C target achievement, finding that only 5.6% of patients met at least one of the recommended targets (7, 14). This is considerably lower than the rates observed in Da Vinci (33%) or Santorini (20.1%) (10, 11), indicating a widening implementation gap.

In subgroup analysis, less than one-third of patients in primary prevention reached LDL-C targets—comparable or lower than Da Vinci results, where 11%–63% of primary prevention patients met their risk-specific goals (10). Notably, 5.7% of DISCOVERY patients with diabetes achieved guideline LDL-C goals, contrasting sharply with 45.5% in the Teresa study (20). The poorest outcomes were seen in patients with ASCVD—only 3.3% reached their LDL-C targets, compared to 18% in Da Vinci (10). This stark discrepancy reinforces the need to address the gap between physician-defined goals and those recommended by guidelines.

Monotherapy was the most common LLT regimen across studies. In Santorini, 54.2% of patients received statin monotherapy and 24% a combination (11); in Da Vinci, moderate-intensity statin monotherapy was dominant (51.8%), and only 9% used statin-ezetimibe combinations (10). Similarly, in DISCOVERY, 47.1% were on moderate-intensity statins and 9.3% on combination therapy (14). Despite an observed increase in rosuvastatin use between visits, dosing remained suboptimal, especially considering that over 60% of patients had ASCVD. High-potency statins were rarely prescribed at dosages sufficient to produce ≥50% LDL-C reduction. Although the use of ezetimibe increased, it remained below 10% at study end, even among ASCVD patients, for whom treatment intensification is crucial given their high residual risk (21, 22). No patients received PCSK9 inhibitors (monoclonal antibodies or siRNA) or bempedoic acid. Across the participating countries, these agents were unavailable or rarely used due to restrictive local reimbursement policies.

The underlying reasons for the disappointing LDL-C target attainment in DISCOVERY are multifactorial. Physician inertia, knowledge gaps, limited confidence in guideline relevance, and a tendency to consider “any statin therapy” sufficient may all contribute. The observed gap between physician-set and guideline-recommended targets is particularly striking, reflecting a mindset where “something is better than nothing” prevails over the evidence-based principle of “the lower, the better.” The physician-set targets likely reflect a range of factors, including clinical judgment, patient-specific considerations, therapeutic inertia, and varying levels of awareness or acceptance of current guidelines. Further research is clearly warranted to better elucidate the underlying factors contributing to these discrepancies, as emphasized in a recent comprehensive review on the topic (23, 24). Addressing these barriers requires a concerted effort to improve physician education, support treatment intensification, and reinforce the importance of LDL-C monitoring and escalation. To overcome therapeutic inertia, low adherence, and barriers within healthcare systems, we propose a multifaceted approach including enhanced patient education and engagement programs, targeted clinician training supported by clinical decision support tools, improved medication accessibility, and the implementation of regular patient monitoring with timely feedback to healthcare providers.

The DISCOVERY study also has limitations. Patient distribution across countries and regions was uneven, which may affect generalizability. The predominance of one region reflects higher site participation and actual population differences. While this may introduce some bias, it also mirrors real-world practice and healthcare structure. Including centers from diverse regions enhances the study's generalizability. The anticipated proportion of patients with incomplete clinical data was 30%, which was closely reflected in the observed rate (31%). This may have contributed to attrition bias. The observational nature of the study may have influenced physician behavior. Adherence data were self-reported and subject to overestimation. The rural–urban patient split was not recorded, and LLT availability varied across countries, possibly affecting consistency. Additionally, the limitations beyond the primary aim and design of our study are the absence of long-term follow-up data and lack of detailed analysis on factors contributing to discrepancies between physicians’ and guideline-recommended LDL-C targets.

In conclusion, the DISCOVERY study provides robust real-world evidence that the 2019 ESC/EAS lipid management guidelines are poorly implemented in countries with high and very high CV risk. Raising awareness of this problem is a critical first step. Based on our findings, a renewed call to action is warranted. Principles such as “decrease as recommended” and “monitor regularly and intensify when necessary” could serve as core messages in a strategic effort to improve lipid management in these regions.

## Data Availability

The datasets generated and analyzed during the current study are available from the corresponding author on request.
