# Supplementary material for: Suboptimal management of hypercholesterolemia in countries with high or very high cardiovascular risk: findings from the international DISCOVERY study
Source: Front Cardiovasc Med. 2025 Sep 5;12:1665059. doi: 10.3389/fcvm.2025.1665059 (PMC12447522; doi:10.3389/fcvm.2025.1665059)
Supplement: Supplementary file 1 [file Datasheet1.pdf]

Supplemental material

Table S1: Acknowledgement

**Center**	**Researcher**
Bosnia and Hercegovina	
UKC Tuzla	Kovačević Maida
PRIV. ORD. AMRA MACIĆ	Macić Džanković Amra
UKC RS	Stanetić Bojan
POLIKLINIKA DR NABIL	Nabil Naser
DZ Zavidovici	Čamdžić Mirsad
DZ Tuzla	Selmanović Senada
JZU SRBIJA	Lalović Ivana
DZ NOVO SARAJEVO	Nadarević Ervin
DZ Laktaši	Panić Bojan
DZ B.Luka	Bijelić Radojka
KB Zenica	Pojskić Belma
DZ Bihać	Sancakli Hodžić Zuhal
PO SUNCE AGRAM	Damjančević Milan
DZ Cazin	Bajramović Zehira
JZU SRBIJA	Pejović Bojan
DZ Lukavac	Osmanbegović Dževdet
DZ Srebrenik	Joldić Belkisa
DZ Srebrenik	Zahirović Emina
DZ B.Luka	Stanetić Kosana
DZ Zenica	Prasko Subhija
MIB	Avdić Sevleta
DZ Prijedor	Todorović Grbić Danijela
OB Doboj	Blagojević Milan
UKC Tuzla	Mršić Denis
DZ B.Luka	Savić Suzana

Internističko kardiološka ordinacija Vitacor	Hajrić Ramiz
KB Bihać	Kurtalić Sadat
Kardiocentar dr Terzić	Terzić Ibrahim
OB Gradiška	Marković Potkonjak Ljiljana
MIB	Osmanović Enes
DZ Zenica	Redžepagić Gavran Larisa
DZ Mostar	Palameta Marnela
SKB Mostar	Brizić Ivica
DZ B.Luka	Radonjić Svjetlana
Kardio centar prof. dr. Zumreta Kušljugić	Kušljugić Zumreta
UKC RS	Kovačević Preradović Tamara
DZ B.Luka	Marković Brankica
KB Zenica	Bajrambašić Emira
DZ Cazin	Mureškić Dževdana
DZ Gračanica	Nukić Sabrija
DZ Zenica	Musić Palalić Indira
DZ Tuzla	Batić Mujanović Olivera
DZ Prijedor	Radanović Gordana
DZ Zenica	Hadžić Verem Anida
DZ Visoko	Alagić Belma
DZ Tuzla	Kamerić Sanadina
DZ ILIJAŠ	Salkić Elvira
DZ NOVI GRAD	Vilić Amela
DZ Tesanj	Dujsić Amir
DZ Gradačac	Tokić Fija
DZ ILIDŽA	Juković Alma
DZ Bihać	Korčić Zemira
UKC Tuzla	Lončar Daniela
DZ Gradačac	Peštalić Mirza

DZ CENTAR	Jatić Zaim
MIB	Djedović Samed
DZ B.Luka	Broćeta Pilipović Nataša
DZ Prijedor	Đerić Dijana
KB Zenica	Pojskić Lamija
OB Prijedor	Lazić Nada
DZ B.Luka	Ruso Zora
UKC RS	Dujaković Boris
DZ B. Luka	Kovačević Irena
DZ NOVO SARAJEVO	Bihorac Kučuk Zumreta
Plava poliklinika Tuzla	Baraković Fahir
DZ NOVO SARAJEVO	Šehović Rasema
DZ Zenica	Gajanović Branka
DZ Čelinac	Kuprešak Draško
DZ Zenica	Muratović Enisa
DZ Bijeljina	Šešlija Ugrinić Maja
KB Zenica	Rošić Almir
DZ Mostar	Zalihić Amra
MIB	Kušljugić Amira
DZ Tuzla	Hajrić Zinaida
OB Bijeljina	Lovrić Milica
DZ STARI GRAD	Ahmetović Samira
DZ NOVO SARAJEVO	Neslanović Abdagić Mubera
DZ Laktaši	Pećanac Žana
PU EMPATHY	Odović Svjetlana
DZ NOVO SARAJEVO	Horman Sanela
DZ Tuzla	Beganlić Azijada
SKB Mostar	Zelenika Diana
DZ B.Luka	Petrović Verica

DZ Srbac	Stojić Mirko
DZ Vogošća	Đurkovski Sandra
DZ Mostar	Černi Obrdalj Edita
DZ Omer Maslić	Toromanović Emra
PO Cardioteam	Kulić Mehmed
DZ Doboj	Vukojević Biljana
OB Prijedor	Vranješ Dalija
OB Prijedor	Graonić Đukić Slađana
DZ ILIDŽA	Osmanagić Mirza
DZ Visoko	Handžić Edina
DZ Doboj	Jotić Ivanović Marina
OB Tesanj	Bedak Omer
DZ Doboj	Nikolić Radoslav
UKCKS	Resić Nerma
DZ ILIDŽA	Fifić Emir
DZ Bihać	Cerić Katmerka
DZ ILIDŽA	Hebibović Ševala
DZ Mostar	Markota Nina
DZ Bijeljina	Vićanović Amela
DZ Vogošća	Duraković-Bejtović Samira
DZ NOVI GRAD	Lihovac Amila
OB Gradiška	Korać Robert
OB Gradiška	Živanić Milanka
DZ NOVI GRAD	Salcer Šunjić Lidija
DZ B.Luka	Stanetić Dijana
DZ Zenica	Mustafić Elida
SKB Mostar	Markota Ivica
UKCKS	Zvizdić Faris
Bolnica Trebinje	Radanović Aleksandar

UKCKS	Šabanović Bajramović Nirvana
UKCKS	Alić Lazović Zina
UKC RS	Dević Željko
UKC RS	Trninić Dijana
DZ NOVI GRAD	Hećo Ljiljana
DZ Srbac	Lepir Dragan
Priv. Odr. Medical Centar Pešto	Pešto Senad
DZ Lukavac	Arapčić Sedija
UKCKS	Sokolović Šekib
DZ Živinice	Jagodić Imamović Fata
UKC RS	Kovačević Siniša
DZ Živinice	Bečirović Duškanka
UKC RS	Kos Ljiljana
DZ Lukavac	Bajrić Samir
DZ Doboj	Popović Željka
DZ V.Kladuša	Mašinović Hadžić Vesna
DZ Saraj polje	Selimović Samira
DZ NOVI GRAD	Eminagić Samra
UKCKS	Iglica Amer
DZ NOVO SARAJEVO	Zećo Emir
UKC Tuzla	Sijerčić Edita
DZ STARI GRAD	Zeković Alma
DZ Bijeljina	Paleksić Dokić Bojana
DZ Bijeljina	Savin Gordana
KB Zenica	Ejubović Malik
UKC Tuzla	Jahić Alan
UKC RS	Gaćanović Žana
OB A. NAKAŠ	Kurbašić Izeta
DZ Tuzla	Mujčinagić Vrabac Muamera

ZU Deamedica BL	Vulić Duško
DZ. Mostar	Sopta Edita
OB Nova Bila	Kolenda Dževada
UKC RS	Živanović Željko
UKC RS	Marjanović Miron
OB A. NAKAŠ	Balijagić Fahrudin
KB Zenica	Štimjanin Ena
UKC Tuzla	Bijedić Irma
DZ NOVI GRAD	Nišić-Redžepagić Refika
OB Doboj	Vasić Nada
SKB Mostar	Avdalović Dejana
DZ Doboj	Tomić Andrea
UKC Tuzla	Bijedić Amira
UKC Tuzla	Dizdarević Hudić Larisa
DZ Trebinje	Bošnjak Tatjana
UKCKS	Hondo Zorica
DZ CENTAR	Hasanović Elvira
UKC RS	Jovanić Jelena
DZ STARI GRAD	Oršolić Gordana
OB Bijeljina	Trifković Mile
UKC RS	Šobot Nikola
UKC RS	Unčanin Dragan
DZ Vogošća	Hamzić Amela
DZ B.Luka	Rupar Verica
OB Prijedor	Dragojević Stojić Maja
DZ Gračanica	Hadžić Jasmin
UKCKS	Hadzibegić Nina
DZ Ključ	Koljić Emil
DZ Trebinje	Babić Aleksandar

PRIV.ORD. KARDIOCENTAR	Čaluk Jasmin
KB Zenica	Mujarić Ekrema
UKC RS	Stojković Sanja
JZU SRBIJA	Kešelj Snježana
UKC RS	Janjić Aleksandar
OB Prijedor	Marin Draženko
DZ Vogošća	Husejnović Čolić Amra
UKC Tuzla	Selimović Mirsad
UKCKS	Hodžić Enisa
UKC Tuzla	Delić Adnan
OB Bijeljina	Janjičić Aleksandar
UKCKS	Durak Nalbantić Azra
DZ Tuzla	Sprečić Vesna
UKCKS	Džubur Alen
UKCKS	Begić Alden
UKC Tuzla	Smajić Elnur
Archive BA	BA Archive
UKCKS	Brigić Čengić Lejla
UKCKS	Jamaković Mesud
UKC Tuzla	Divković Katarina
Bulgaria	
AIPPMP- D-R MARIA MILEVA-KUMCHEVA EOOD	Kostadinova Mileva-Kumcheva Maria
ASMP-IPK	Rumenov Rusanov Angel
ASIMPK -IP D-r Ralica Tchavdarova	Georgieva Ralica
AISMP D-r Daniel Trendafilov	Peykov Trendafilov Daniel
ET Medika -66 d-r Mitko Hadjiev APMP-IP	Tsenkov Hadzhiev Mitko
AGPPMP Purva lekarska praktika OOD	Nikolaev Kalaydzhiev Aleksandar
AIPMP - IPPMP D-R TIHOMIRA ZLATANOVA Ltd	Zlatanova Zlatanova Tihomira
MC SANA 3 OOD Andrey Aleksandrov Andreev	Aleksandrov Andreev Andrey

D-r Anatoli Tachov -IPSMP in internal diseases and cardiologyEOOD	Mihaylov Tachov Anatoli
D-r Todorovi - GPPMP OOD	Dinov Todorov Veselin
DCC 1 Varna	Nikolov Kostadinov Atanas
AIPPMP D-r Desislava Kovacheva	Georgieva Kovacheva Valeva Desislava
AIPPMP D-r Elenski EOOD	Hristeva Foreva Gergana
Second Medical Center - Sofia	Vasilev Todorov Georgi
Medicos OOD	Miteva Gencheva Denka
DKC-2 Pleven-EOOD	Velikova Diana
AIPPMP - D-R A. STRUMELIEVA EOOD	Ivanova Strumelieva-Tancheva Antoaneta
DR SLAVI SLAVOV IPPMP 2002 EOOD	Atanasov Slavov Slavi
AIPPMP D-r Anton Donchev	Ivanov Donchev Anton
DKC Avis Medika	Bojidarova Bojadgieva-Marincheva Desislava
D-r Asen Ivanov Borisov APMP-IPPMP	Ivanov Borisov Asen
Medicus Stars Ltd.	Ivanov Valov Steliyan
ET D-r Mariq Raicheva AIPMP	Raycheva Raycheva Marija
ET D-r Nadya Andreeva -Edreva AIPPMP	Petkova Andreeva-Edreva Nadya
GPPMP VITA 2002	Yankov Stojanov Nikolay
D-r Ekaterina Lulova - IPSMP in Internal diseases and Cardiology EOOD	Kostadinova Lulova Ekaterina
APIMP-IP	Angelova Nakova Petya
IPPMP Megamed LTD	Vyleva Vrajilova Daniela
Doktor Branimir Belishki AIPPMP-Fiora EOOD	Vladimirov Belishki Branimir
Medikus,d-r Pepa Ganchevska Ferreira	Ganchevska Ferreira Pepa
D-R Dimitur Hadzhiev-IPSMP in cardiology EOOD	Yordanov Hadzhiev Dimitar
DCC 1 Varna	Hayredinova Hadzhieva Birsen
IPPIMP-D-r Petko Zhelyazkov	Zhelyazkov Petko
MC Orbita - Ivo Ralchovski	Petrova Yordanova Petya

ASIMPK -IP	Valkova Georgieva Galina
D-r Gatev i D-r Mihaylova-GPPIMP OOD	Georgieva Mihaylova-Gateva Slavina
D-R DZHAMBOVA-AIPPMP-PLOVDIV EOOD	Rangelova Dzhambova Pavlina
STANCHEV MEDICAL AIPPMP EOOD	Atanasov Stanchev Veselin
GPPMPAktamed	Petkov Peshkov Ognyan
HIGIA 2 - AGPPMP OOD	Yordanova Dobreva-Pavlova Vanya
HELT 94 AIPIPMP EOOD	Todorova Lazarova Zlatka
D-R MINASYAN - AIPPMP EOOD	Hachik Minasyan-Encheva Petlehem
DKC Tchaika	Nikolov Boyadzhiev Vladimir
STANIVA AIPIPMP EOOD	Vasileva Ivanova-Zapryanova Stanimira
IP Georgieva	Mihailova Ralcheva-Georgieva Violeta
ET Vanya Raycheva - ASMP - IPSMP Cardiologiya	Raycheva Valkova Vanya
MC Yanev, Varna	Rumenov Yanev Stefan
Familna medicina-S-GP-PMP OOD	Stoychev Stanoev Ivan
D-R PENKO ZAPRYANOV - APMP-IP EOOD	Kostadinov Zapryanov Penko
D-R PETYA TODOROVA MADZHAROVA - APIMP-IP EOOD	Todorova Madzharova Petya
D-r Yusevi-AGPPIMP OOD	Yasenova Yuseva Nina
IPPMP DR DJENYO DZHENEV EOOD	Atanasov Dzhenev Djenyo
AIPPMP - D-R GEORGI TSIGAROVSKI EOOD	Ivanov Tsigarovski Georgi
ET D-r Cvetan Ruskovski AIPPMP	Dimitrov Ruskovski Tsvetan
ET D-R NEDKA STAYKOVA-STOYANOVA - AIPPMP	Staykova Staykova-Stoyanova Nedka
ET AIPPIMP - d-r Seslav Stefanov	Nikolaev Stefanov Seslav
ET APIMP D-r Veselina Trifonova	Trifonova Vasileva Veselina
ET dr. Reni Patarinska APMPIP	Ivanova Patarinska Reni
Sv.Sv. Konstantin i Elena - GPPMP OOD	Atanasov Kulinski Krasimir
AIPSMP	Cvetanov Yanchev Nikola
MC Arcus Medikul	Stefanova Ignatova Daniela
Meditsinski tsentar po prevantivna cardiologiya - SV. Ivan Rilski Chudotvorets EOOD	Mladenov Rusinov Veselin

MC Kalimat	Dragoitchev Cvetkov Krassimir
Medical Center-Ruse EOOD	Kalev Dimitrov Stoil
AIPSMPK D-r Ilija Janev	Iliya Yanev
MC Nevromediks	Vasileva-Boyadzhieva Liliya
ASMP D-R RUMYANA STOYCHEVA VASILEVA EOOD	STOYCHEVA VASILEVA RUMYANA
DCC III - Sofia	Petkova Ilieva - Slavcheva Radostina
Medical centar East	Dimitrov Doychinov Rosen
DKC PLOVDIV EOOD	Petkova Mustakova-Kostova Spaska
DCC Sv. Anna EOOD	Plamenova Karatancheva Blagorodna
DKC 1 Kardzhali OOD	Aldinov Hadzhiev Pavel
ASIMPKIP-D-R ELENA DOTCHEVA-EOOD	ATANASOVA DOTCHEVA ELENA
Medical center 1 Harmanli EOOD	Lilyanova Valkova Yuliyana
IPSMP D-r Nevena Karcheva EOOD	Georgieva Karcheva Nevena
AIPSIMPK - Tonus - dr. Nora Milcheva EOOD	Petrova Milcheva Nora
IPSPM PO KARDIOLOGIYA D-R YULIYANA TONCHEVA EOOD	Toncheva Ivanova Yuliyana
Medical Center Cardiomed	Lavila Kamara Daniek
MBAL TRAKIA PARK	Stefanov Banov Todor
DKC BURGASMED EOOD	NIKOLOVSKI BORCHE
DKC Karidad - Asenovgrad	Borisov Tyutyundzhiev Stefan
DKC Sveti Georgi EOOD	Shukri Dural Meral
Medical center I- Asenovgrad EOOD	Gancheva Ganeva Katya
MC Plazmamed, Varna, ul. Aleksandar Dyakovich 31	Rushid Mesut
St.Marina Medical Center-Pleven	Ognyanova Petsova-Kuzmanova Teodora
D-r Orlin Cvetkov-cardiolog-AIPSMP EOOD	Cvetkov Orlin
DCC Sveta Marina Varna, bul. Hristo Smirnenski 1	Yordanov Rumen
MEDICAL CENTER FOR LIFE EOOD	ISMEDOV MEHMEDOV ISMED
Cardis Dr Lyudmila Ivanova Gotseva AIPSMP-VBK	Gotseva Lyudmila
MC D-R DIMITROVI EOOD	GOSPODINOV DIMITROV STEFAN

ASMP - IPSMP - Cardiologiya - dr. Venetsio Netsov EOOD	Netsov Venetsio
MC Sartse - 21 vek OOD	Kamenov Dimitar
MBAL BEROE	Svetozarov Leshtevski Vasil
Medical center Noleks OOD	Petrova Pisanova- Eftimova Anastasiya
DKC Veliko Tyrnovo	Avramchev Petyo
DCC2-Ruse	Georgiev Pavlov Stanislav
GPSMP-kardiologiya Zdrave i zivot EOOD	Vergilov Kolomanov Borislav
UMHAT Dr. Georgi Stranski	Yuliyanova Stancheva- Hristova Nadya
Medical Center I Stamboliyski	Myumyun Adem Selvinaz
D-R GEORGI GYUZELEV-IPSMP EOOD	GYUZELEV Georgi
Medical Center HBO2	Egeniev Borisov Radoslaw
DKC -Smolyan EOOD	Kovacheva-Dascalova Zinaida
DKC-Smolyan EOOD	Georgieva Yocheva Siyka
MC Zdrave - Lom EOOD	ZHIKOV TRIFONOV NIKOLAY
AISMP (Cardiologica doctor Aleksiev) EOOD	Aleksiev Aleksandar
GPSMP Krasteva i Kadiyski OOD	Krasteva Nina
Medical Center Panteleymon	Vasilev Zhykov Nikolay
IPSMPK dr Ivan Gerchev EOOD	Petkov Gerchev Ivan
ASMP IP D-R OGNIAN ILIEV EOOD	Iliev Ognian
MC Yoan Pavel II EOOD	Savova-Petkova Detelina
DKC 1, Stara Zagora	Angelov Minkov Plamen
ASIMPVBK IP Cardio Tonus EOOD	Petrov Ivanov Ivaylo
Hungary	
Private practice HU	Nagy Péter
Private practice HU	Ábrahám Judit
Private practice HU	Jerkovits Gergely
Private practice HU	Bojtor Éva
Private practice HU	Huszár Erika Ágnes
Private practice HU	Fodor Éva Anna

Private practice HU	Szödényi Annamária
Private practice HU	Mihályi Zsuzsanna
Private practice HU	Zuberecz Zoltán
Private practice HU	Győriné Mező Beáta
Private practice HU	Várkonyi Katalin
Private practice HU	Takács Ágnes
Private practice HU	Kohári György
Private practice HU	Tóth Éva
Private practice HU	Nagy Éva
Private practice HU	Szauter Csaba Gábor
Private practice HU	Berki Barnabás
Private practice HU	Szittyai Borbála
Private practice HU	Bauer Kálmán
Private practice HU	Ambus Anikó
Private practice HU	Belák Márk
Private practice HU	Antunoviics Helga
Private practice HU	Jánovszky Zita
Private practice HU	Török Katalin
Private practice HU	Katalin Dán
Private practice HU	Zoltán Fónagy Sütő
Private practice HU	Gyöngyi Kosits
Private practice HU	Papp Albert
Private practice HU	Bíró Attila
Private practice HU	Tompos Gábor
Private practice HU	Andrea Kis
Private practice HU	Handl Mária
Private practice HU	Varga Irma Ildikó
Private practice HU	Dobó Éva
Private practice HU	Magyar László

Private practice HU	Mohos András
Private practice HU	Simon Tünde
Kosovo	
University Clinical Center of Kosovo	Pllana Edita
Regional Hospital	Ademi Dergut
Regional Hospital	Hajdari Halim
Regional Hospital	Nalbani Ali
University Clinical Center of Kosovo	Qitaku Hajdin
University Clinical Center of Kosovo	Ahmeti Artan
University Clinical Center of Kosovo	Pllana Gojart
Regional Hospital	Prenaj Agron
University Clinical Center of Kosovo	Hima Fisnik
Regional Hospital	Kongjeli Arta
Regional Hospital	Mehmeti Naza
Regional Hospital	Muhaxheri Elfedin
Regional Hospital	Kryeziu Milazim
Regional Hospital	Bajrami Raim
Regional Hospital	Blakaj Edita
Regional Hospital	Berisha Galina
Regional Hospital	Keka Zenun
University Clinical Center of Kosovo	Haliti Edmond
Regional Hospital	Rahmani Remzi
Regional Hospital	Pllana Bujar
Regional Hospital	Berisha Gezim
University Clinical Center of Kosovo	Mahmutaj Vigan
University Clinical Center of Kosovo	Batalli Arlind
Regional Hospital	Hasani Bashkim
University Clinical Center of Kosovo	Bajraktari Gani
Moldova	

National Cardio Institute, Chisinau	Moiseeva Anna
Archive MD	Archive MD
National Cardio Institute, Chisinau	Sapojnic Nadejda
National Cardio Institute, Chisinau	Bitca Angela
North Macedonia	
ЈЗУ Општа болница Охрид	Василевски Сашо
ЈЗУ Општа болница Струмица	Христов Гоце
ЈЗУ Специјализирана болница за превенција, лекување и рехабилитација на кардиоваскуларни заболувања	Митревски Стојан
Поликлиника МВР	Секованиќ Љопче
Здравствен дом Скопје Поликлиника Идадија	Иванов Љупчо
ЈЗУ Специјализирана болница за превенција, лекување и рехабилитација на кардиоваскуларни заболувања	Рилинд Жаку
ПЗУ Гастротоп	Новевска Ѓоршовска Љубица
ЈЗУ Клиничка болница Тетово	Ајрули Надир
ЈЗУ Клиничка болница Тетово	Стефановска Душица
ПЗУ Др. Христина	Лескароска Христина
ПЗУ Самарџиски	Трајчевска Симона
ГОБ 8ми Септември	Албарагони Самир
ПЗУ Др. Каракотев	Каракотев Ѓорѓи
ЈЗУ Општа болница Гевгелија	Чочков Благој
Здравствен дом Скопје Поликлиника Битпазар	Аврамовски Борис
ПЗУ Интергин	Зарковска Оливера
ЈЗУ Клиничка болница Тетово	Асани Валон
ЈЗУ Општа болница со проширена дејност Кавадарци	Митева Роза
ПЗУ Акус Медикус	Крстевски Дејан
ЈЗУ Универзитетска клиника за кардиологија	Костов Јорго
Здравствен дом на железничарите, Поликлиника Железничар	Двојакова - Маркоски Јасна
ЈЗУ Клиничка болница Тетово	Мамути Бекир

ЈЗУ Општа болница Струга	Костојчиноска Мира
ПЗУ Др. Златко Стојменов	Димитровски Александар
ПЗУ Дијагностички центар	Колева Билјанка
ЈЗУ Клиничка болница Тетово	Абази Неџбедин
Универзитетска клиника за хируршки болести „Св. Наум Охридски“	Отљански Александар
ПЗУ Мултимедика Интерна	Михајловски Зоран
ЈЗУ Општа болница Гостивар	Нуредини Линдита
ЈЗУ Општа болница Гостивар	Сулејмани Гојарт
Здравствен дом Крива Паланка	Николовски Сретимир
ПЗУ Санитас	Солтировска Ирена
ЈЗУ Специјализирана болница за нефрологија	Стојаноска Татјана
ПЗУ Др. Златко Стојменов	Коцев Ванчо
ЈЗУ Општа болница Гостивар	Весели Абази Алсада
ЈЗУ Специјализирана болница за превенција, лекување и рехабилитација на кардиоваскуларни заболувања	Цаноска Танеска Наташа
ЈЗУ Општа болница Прилеп	Савевски Златко
Клиничка болница Аџибадем Систина	Слободан Антов
Здравствен дом Скопје Поликлиника Битпазар	Блажевска Тасеска Даниела
ЈЗУ Универзитетска клиника за кардиологија	Митевска Ирена
Поликлиника Јане Сандански	Арсиќ Оливера
ЈЗУ Универзитетска клиника за кардиологија	Костова Нела
ПЗУ Манолеви	Панделиев Никола
ЈЗУ Општа болница Гостивар	Бајрами Емри
ЈЗУ Општа болница Куманово	Бајдевска Спирковска Марија
ЈЗУ Општа болница со проширена дејност Кавадарци	Маневски Дукадин
ЈЗУ Универзитетска клиника за кардиологија	Петкоска Спирова Даница
ГОБ 8ми Септември	Симоновски Кочо
Здравствен дом Поликлиника Ѓорче Петров	Пашоска Тереза

ПЗУ Панови	Спасов Даниел
Клиника Жан Митрев	Идризи Шпенд
ЈЗУ Специјализирана болница за нефрологија	Цапоска Олга
ЈЗУ Специјализирана болница за превенција, лекување и рехабилитација на кардиоваскуларни заболувања	Танески Филип
ЈЗУ Универзитетска клиника за кардиологија	Попоска Лидија
Здравствен дом Скопје Поликлиника Идадија	Корнети Благица
ЈЗУ Општа болница Охрид	Целеска Виолета
Поликлиника Јане Сандански	Лакинска Славица
ЈЗУ Универзитетска клиника за кардиологија	Бошев Марјан
ПЗУ Цитус	Мартиноска Андонова Зорица
ЈЗУ Општа болница Прилеп	Милевска Александра
ЈЗУ Општа болница Струмица	Иванов Методи
Здравствен дом Скопје Поликлиника Чаир	Симјановска Анита
ЈЗУ Општа болница Кичево	Сејдини Арлинд
ПЗУ Неуромедика	Јанковски Жарко
Здравствен дом Поликлиника Драчево	Димитрова Чагановиќ Викторија
ЈЗУ Клиничка болница Штип	Докузова Стојка
ЈЗУ Општа болница Гостивар	Бошкоски Стојо
ЈЗУ Универзитетска клиника за кардиологија	Чапароска Емилија
ПЗУ ДР. Ацо Николов	Поповска Верица
ЈЗУ Клиничка болница Штип	Јорданова Славица
ПЗУ Србиновски	Богевска Блажевска Митра
ЈЗУ Универзитетска клиника за кардиологија	Талески Јане
ЈЗУ Универзитетска клиника за кардиологија	Јовковски Александар
ГОБ 8ми Септември	Неческа Елена
ЈЗУ Универзитетска клиника за кардиологија	Шеху Енес
ПЗУ Др. Горан Денковски	Бошковски Мирослав
ЈЗУ Општа болница Куманово	Радончиќ Лејла

ПЗУ Медикор	Топаловски Виктор
ЈЗУ Клиничка болница Тетово	Кикирковска Елена
ПЗУ Дарон	Цветаноска Марина
ЈЗУ Клиничка болница Тетово	Ферати Фатмир
ЈЗУ Универзитетска клиника за кардиологија	Паљошковска Јорданова Саветка
ЈЗУ Клиничка болница Штип	Накова Валентина
ЈЗУ Универзитетска клиника за кардиологија	Зафировска - Талеска Биљана
ЈЗУ Клиничка болница Тетово	Шабани Јусуф
ГОБ 8ми Септември	Јованоски Златко
Клиничка болница Битола	Ивановска Елизабета
Клиничка болница Битола	Трајковски Боби
ЈЗУ Општа болница Охрид	Цуцулоски Томислав
ЈЗУ Клиничка болница Штип	Ушинова Весна
ЈЗУ Општа болница Кочани	Јакимова Верица
ЈЗУ Клиничка болница Тетово	Ангелеска Јорданка
ЈЗУ Универзитетска клиника за кардиологија	Ристески Дејан
ЈЗУ Клиничка болница Штип	Јовев Марјан
ПЗУ Др. Јанев	Јанев Тони
ЈЗУ Специјализирана болница за нефрологија	КоваческаВанѓелка
ЈЗУ Општа болница со проширена дејност Кавадарци	Ридов Еленчо
Клиничка болница Битола	Вељановска Ана
Клиничка болница Битола	Ерка Теодор
ЈЗУ Специјализирана болница за превенција, лекување и рехабилитација на кардиоваскуларни заболувања	Тупаре Слаѓана
Клиничка болница Битола	Митревски Јован
Здравствен дом Свети Николе	Панева Славица
Клиничка болница Битола	Лозанче Ненси
ЈЗУ Универзитетска клиника за кардиологија	Јанушевски Филип
Здравствен дом Вевчани	Демишоски Аладин

ПЗУ Ехо медика	Галачев Димитар
ПЗУ Србиновски	Шишкова Билјана
ПЗУ Кардиолек	Шопов Киро
ЈЗУ Клиничка болница Штип	Василев Благој
ЈЗУ Специјализирана болница за превенција, лекување и рехабилитација на кардиоваскуларни заболувања	Донески Зоран
ЈЗУ Специјализирана болница за превенција, лекување и рехабилитација на кардиоваскуларни заболувања	Фортомароска Благовесна
ПЗУ Медикус 92	Волчева Билјана
ЈЗУ Општа болница Охрид	Милошеска Валентина
ЈЗУ Клиничка болница Тетово	Трајчески Тед
ЈЗУ Универзитетска клиника за кардиологија	Арнаудова Фросина
ЈЗУ Универзитетска клиника за кардиологија	Поцеста Беким
ПЗУ Дарон	Наумоски Јане
ЈЗУ Специјализирана болница за превенција, лекување и рехабилитација на кардиоваскуларни заболувања	Секулоски Ристе
ЈЗУ Универзитетска клиника за кардиологија	Пејков Христо
ЈЗУ Општа болница Струга	Кузмановска Билјана
ЈЗУ Општа болница Гостивар	Даути Бетим
ЈЗУ Специјализирана болница за нефрологија	Деребанова Ана
ПЗУ Др. Валентина Рамбабова	Крстева Тања
ЈЗУ Универзитетска клиника за кардиологија	Китановски Дарко
Клиника Жан Митрев	Нешковски Илија
ПЗУ Интер ехо	Галев Трајче
Клиника Жан Митрев	Ѓоргов Никола
ЈЗУ Универзитетска клиника за кардиологија	Петрески Живко
Клиничка болница Аџибадем Систина	Душкоски Димитар
ЈЗУ Универзитетска клиника за кардиологија	Таравари Хајбер
ЈЗУ Општа болница Гевгелија	Мат Патрик

Mongolia	
Health center of Sukhbaatar	Adiyasuren Shurentsetseg
Health center of Bayangol	Erdenetsog Enkhjargal
3rd clinic	Tsembel Tsolmon
Health center of Uvs province	Tulga Amarzaya
Health center of Sukhbaatar province	Batkhuu Urangoo
Health center of Zavkhan province	Byambajav Puntsagdulam
Erdenet medical diagnostic and treatment center	Adilbish Undrakh
Health center of Bayanzurh	Dagiiragchaa Unursaikhan
Health center of Baganuur	Lkhagvasuren Chimedlkham
Clinic Grandmed	Uugantsetseg Byambatsogt
Romania	
BC	Afemei-Pascaru Cristina
B2	Alexandrescu Gheorghe
CJ	Andreiu Edit
B3	Aron Ramona
AG	Atanasiu Marius
B3	Bădescu Elena
GL	Baiesiu Simona Alina
CJ	Bardos Kinga Tunde
CT	Belciug Mirela
AG	Berbece Elena Claudia
B2	Berbecel Cristina Catinca
MS	Bernea Sorin
TM	Birou Liliana
BC	Blaga Angelica
B2	Boboc Valentina
BC	Boldisor Cristina
GL	Borbil Gabriela

PH-BV	Brîndușoiu Laurențiu
B2	Burducea Anca
B2	Buta Fanica
IS	Calarasu Adrian Cezar
CJ	Caloian Bogdan
PH-BV	Chiriță Rodica
CJ	Chkess Liliana
SB	Cioran Nicoleta
CJ	Comsa Horatiu
AG	Corina Popa
B2	Cornea Ana Delia
IS	Corodescu Lavinia
SB	Costea Alexandru
BC	Costin Lucia
OT	Cristina Luba
IS	Daminescu Irina
B2	Dinu Iulian
B1	Dodoiu Sergiu
BH	Dragomir Dinu Andrei
BH	Dragusan Ana Maria
CJ	Dron Camelia
TM	Duda Seiman Daniel Marius
B2	Dumitrescu Dragos
B1	Dumitrescu Elena
OT	Elena Gogonea
B1	Feidoc Roxana
CT	Filip Nadina
PH-BV	Gabor Anca Laura
PH-BV	Găjman Doru

B3	Geiculescu Călin Mihai
GL	Ghenadi Alexandru
GL	Ghenadi Claudia
B3	Gheorghe Alina Iuliana
GL	Goldura Ionel
GL	Goldura Neguta
B2	Grama Lavinia
TM	Gruici Adrian-Nicolae
GL	Guzga Cristina
GL	Harpalete Angela
GL	Hodorogea Camelia
MS	Horga Diana
PH-BV	Hrubaru Răzvan
TM	Huh Mihaela
TM	Ianos Raluca
PH-BV	Ioniță Silvian
CJ	Jecan Voichita
IS	Licau Daniela
B1	Licudis Paula
MS	Maftey Judit
CJ	Magurean Simona
OT	Manea Alexandru
TM	Mates Adela Sofia
B1	Mesaros Bogdan
BH	Mester Andras
PH-BV	Mihăilescu Corina
B1	Mihiotis Silvia
B3	Mincinoiu Viorel
TM	Mogosanu Oana

B2	Moise Sanziana
MS	Morariu Mirabela
CJ	Mustacila Adrian Gabriel
SB	Neamtiu Ileana
TM	Neamtu Simina
BH	Nichita Brendea Mihnea Traian
SB	Nicola Eniko
CT	Oloeanu Marinela
B3	Olteanu Ilona Carmela
B3	Oprișan Anca
BH	Oradea Cozma Alexandra
B2	Pana Monica Elena
TM	Pătrău Elena
B2	Pelican Mariana
B1	Petre Ionut Catalin
PH-BV	Popescu Dorin
B3	Popescu Mariana
BC	Preda Mariana
PH-BV	Prică Sorin
B3	Prundeanu Luminita Roxana
SB	Ramona Teodora Rau
CJ	Rogojan Maria Stela
GL	Ropotan Alexandra
IS	Rosca Irina
CJ	Rusneac Dorina
CJ	Sasa Mihaela
AG	State Ieremia Raluca Alexandra
B2	Stefanescu Eugenia
B3	Ștefănescu Silvia

B2	Stoian Marilena
CT	Tache Traian
BH	Tapos Adrian
BH	TOLNAI ANGELA
SB	Vasilache Veronica
OT	Veleanu Liana
PH-BV	Vlăsceanu Ioana
GL	Voda Raluca
B1	Voican Teodor
B3	Voicescu Antonia
AG	Voinea Stefan Valeria
TM	Zarici Iuliana
CJ	Zeicu Adrian
MS	Zoltan Szabo
Slovenia	
Ambulanta splošne medicine Alenka Simonič, dr. med., Ormož	Simonič Alenka
Tomaž Schaubach zasebni zdravnik druž. med., Trnovska vas	Emeršič Ines
Zdravstveni dom Celje	Bračun Ivanka
Splošna bolnišnica dr. Jožeta Potrča Ptuj	Cenčič Vid
Zdravilišče Rogaška - Zdravstvo d.o.o.	Jazbec Čoh Metka
Zdravstveni dom Ilirska Bistrica	Tomić Sunčana Marija
MC Krka	Legiša Simona
Ambulanta Dolenc, d.o.o., Škofja Loka	Godec Darjan
Zdravstveni dom Slovenska Bistrica	Pravdič Urška
Zdravstveni dom Ljubljana	Strgar Hladnik Mihaela
Zdravstveni dom Ivančna Gorica	Pivk Lana
Zas. amb. Sarajlič Amna dr. med. spec.	Sarajlić Amna
Dentiko, d.o.o., Splošna ambulanta Arena	Nagy Marijana
Zdrav slog, d.o.o	Rebol Zadravec Mojca

Zdravstveni dom Šentjur pri Celju	Zupanc Darja
Zdravstveni dom Krško	Cepić Branka
Zdravstveni dom Nova Gorica	Princes Blaž
Zdravstveni dom Velenje	Brunšek Strašek Marta
Zasebna splošna ambulanta Novak	Novak Jasna
Zasebna ambulanta za spl. medicino in akupunkturo Fürst Karmen, dr. med.	Fürst Karmen
Zdravstveni dom Ormož	Ferjuc Anita
Divina Medica d.o.o.	Križevnik Lucija
Zasebna ambulanta Kralj dr. Roman	Kralj Roman
Ambulanta družinske medicine Melanija Nikič Gačeša	Nikić Gačeša Melanija
Zdravstveni dom Šmarje Pri Jelšah	Kostić Dijana
Zdravstveni dom Grosuplje	Dolinar Janko
Ahlin Dragotin-splošna ambulanta	Ahlin Dragotin
Splošna bolnišnica dr. Jožeta Potrča Ptuj	Pagliaruzzi Miha
Medicina Iljaž, d.o.o., Brežice	Iljaž Rade
Splošna bolnišnica dr. Jožeta Potrča Ptuj	Horvat Primož
Zdravstveni dom Ormož	Hekić Goran
Zdravstveni dom Grosuplje	Voga Denis
Zdravstveni dom Radlje ob Dravi	Novak Miro
Zasebna ambulanta Remedia	Štefančič Gašperšič Marija
Zdravstveni dom Sevnica	Jelovčan Ivana
Zdravstveni dom Sežana	Maraž Neda
Zdravstveni dom Laško	Strel Bilka
Zdravstveni dom Nova Gorica	Ahačič Srednik Nika
Zdravstveni dom Ivančna Gorica	Plut Švigelj Mateja
Zdravstveni dom Kamnik	Trunk Judita
Zdravstveni dom Gornja Radgona	Halas Nejc
Zasebni Zdravstveni zavod Bossman Peter	Bossman Peter

MEDICA PRIMA d.o.o	Šimenko Barbara
Zdravstveni dom Koper	Zadravec Radi Andreja
Zasebna ambulanta Aleš Blaznik, dr. med.	Blaznik Aleš
Ambulanta splošne medicine Majda Ambrož Mihelčič, dr. med., Kamnik	Ambrož Mihelčič Majda
Zdravstveni dom Brežice	Pibernik Mojca
Zdravstveni dom Ribnica	Dražetič Nives
Zas. amb. splošne medicine, Ljiljana Todorović Popović, dr. med.	Todorović Popović Ljiljana
Zdravstveni zavod družinska medicina Nova Gorica	Komel Jakob
Zdravstveni dom Cerknica	Doles Olga
Zdravstveni dom Žalec	Ferant Žan
ŽIVA V PARKU, zdravstvene storitve d.o.o.	Cvek Nina
Zdravstveni dom Ilirska Bistrica	Vinšek Grilj Andreja
Zdravstveni dom Domžale	Burja Polona Darja
Zdravstveni zavod dr. Vučko	Vučko Gregor
Zdravstveni dom Žalec	Oblak Mateja
Zdravstveni dom Nova Gorica	Dolinar Kante Helena
Zas. amb. Tenyi dr. Laszlo	Tenyi Laszlo
Zdravstveni dom Murska Sobota	Köveš Leon
Zdravstveni dom Krško	Kus Sotošek Ingrid
Zdravstveni dom Šentjur pri Celju	Podgoršek Estera
Družinska medicina Žagar, d.o.o.	Smrzlić Slađana
Zdravilišče Rogaška - Zdravstvo d.o.o.	Žohar Petra
Zdravstveni dom Ribnica	Dražetič Nives
Zdravstveni dom Sevnica	Malešič Karin
Zasebna ambulanta družinske medicine s koncesijo Jurij Pesjak, dr. med., Sevnica	Pesjak Jurij

Table S2: 2019 ESC/EAS risk-based LDL-C goals

	**Definition of risk**	**LDL-C target goal**
Very high risk	• Documented ASCVD, either clinical or unequivocal on imaging (i.e. previous ACS, stable angina, coronary revascularization, stroke and transient ischaemic attack, and peripheral arterial disease. Unequivocally documented ASCVD on imaging includes those findings that are known to be predictive of clinical events, such as significant plaque on coronary angiography or CT scan defined by multivessel coronary disease with two major epicardial arteries having >50% stenosis, or on carotid ultrasound.<br>• Diabetes mellitus (DM) with target organ damage, ≥3 major risk factors, or early onset of type 1 DM of long duration (>20 years).<br>• Severe chronic kidney disease (eGFR <30 mL/min/1.73 m^2)<br>• Calculated SCORE ≥10% for 10-year risk of fatal CVD.<br>• FH with ASCVD or with another major risk factor.	A therapeutic regimen that achieves ≥50% LDL-C reduction from baseline and an LDL-C goal of <1.4 mmol/L (<55 mg/dL).

	Definition of risk	LDL-C target goal
High risk	• Markedly elevated single risk factors, in particular total cholesterol >8 mmol/L (>310 mg/dL), LDL-C >4.9 mmol/L (>190 mg/dL), or blood pressure ≥180/110 mmHg.<br>• Patients with FH without other major risk factors.<br>• Patients with DM without target organ damage*, with DM duration ≥10 years or another additional risk factors.<br>• Moderate CKD (eGFR 30–59 mL/min/1.73 m^2).<br>• A calculated SCORE ≥5% and <10% for 10-year risk of fatal CVD.	A therapeutic regimen that achieves ≥50% LDL-C reduction from baseline and an LDL-C goal of <1.8 mmol/L (<70 mg/dL).
Moderate risk	• Young patients (T1DM <35 years; T2DM <50 years) with DM duration <10 years, without other risk factors.<br>Calculated SCORE ≥1% and <5% for 10-year risk of fatal CVD.	A goal of <2.6 mmol/L (<100 mg/dL).
Low risk	• Calculated SCORE <1% for 10-years risk of fatal CVD.	A goal of <3.0 mmol/L (<116 mg/dL).

LDL-C - LDL-cholesterol; ASCVD - atherosclerotic cardiovascular disease; CV(D) - cardiovascular (disease); FH - familiar hypercholesterolemia; DM - diabetes mellitus; SCORE - Systematic Coronary Risk Evaluation.

*Target organ damage is defined as microalbuminuria, retinopathy, or neuropathy.

Figure legend

Figure S1:Target LDL-C levels based on 2019 ESC/EAS Guidelines for the management of dyslipidemias and factors for defining the cardiovascular risk of different groups of patients with hyperlipidemia based on 2019 ESC/EAS Guidelines for the management of dyslipidemias and 2021 ESC Guidelines on CVD prevention in clinical practice.

Figure

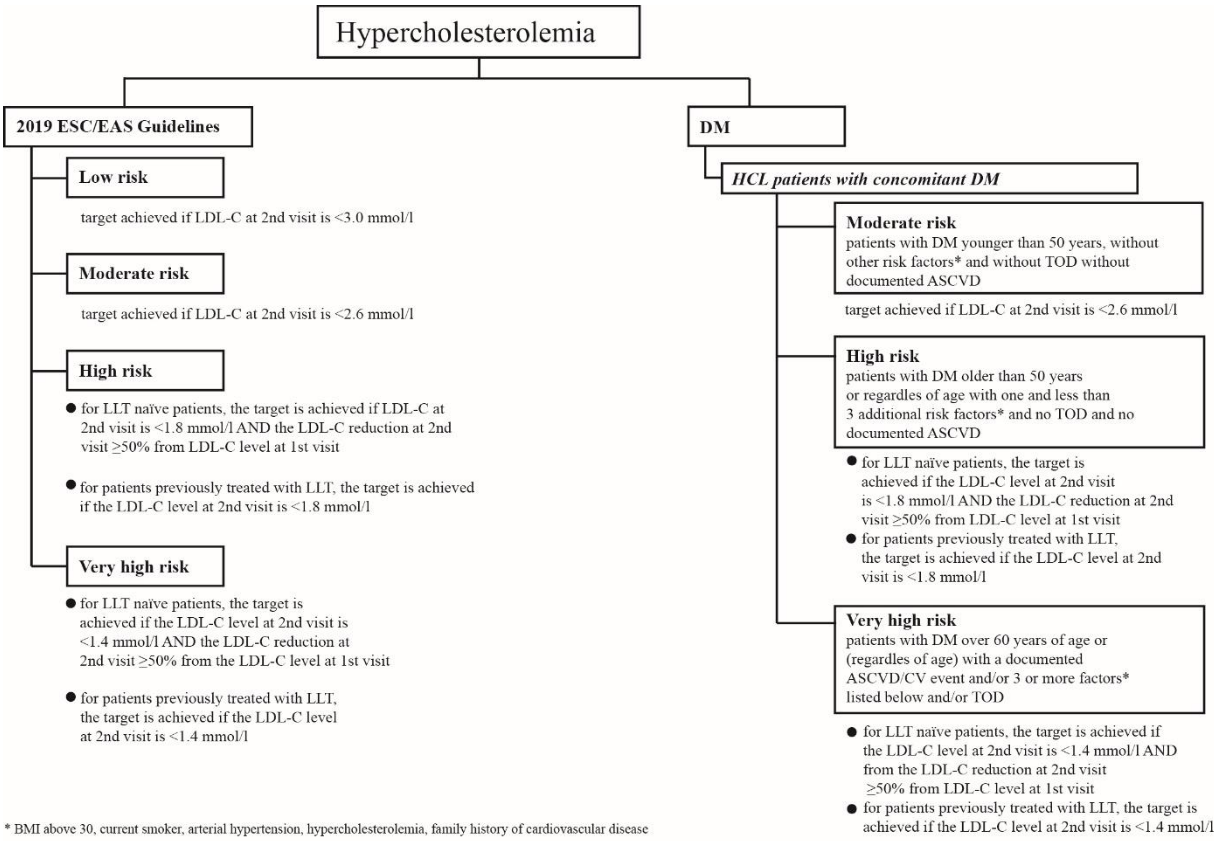

Figure S1: Target LDL-C levels based on 2019 ESC/EAS Guidelines for the management of dyslipidemias and factors for defining the cardiovascular risk of different groups of patients with hyperlipidemia based on 2019 ESC/EAS Guidelines for the management of dyslipidemias and 2021 ESC Guidelines on CVD prevention in clinical practice

HCL – hypercholesterolemia; DM – diabetes mellitus; LDL-C – LDL-cholesterol; (AS)CV(D) – (atherosclerotic) cardiovascular (disease); LLT – lipid-lowering therapy; TOD – target organ damage
